# Electrochemical, Tribological and Biocompatible Performance of Electron Beam Modified and Coated Ti6Al4V Alloy

**DOI:** 10.3390/ijms22126369

**Published:** 2021-06-14

**Authors:** Maria Nikolova, Maria Ormanova, Veselina Nikolova, Margarita D. Apostolova

**Affiliations:** 1 Department of Material Science and Technology, University of Ruse “A. Kanchev”, 7017 Ruse, Bulgaria; 2 Institute of Electronics Acad. E. Djakov, Bulgarian Academy of Sciences, 1784 Sofia, Bulgaria; maria.ormanova@abv.bg; 3 Medical and Biological Research Laboratory, Roumen Tsanev Institute of Molecular Biology, Bulgarian Academy of Sciences, 1113 Sofia, Bulgaria; vnikolova@bio21.bas.bg (V.N.); margo@bio21.bas.bg (M.D.A.)

**Keywords:** surface patterning, electron beam modification, roughness, TiN/TiO_2_ coating, osteogenic cells

## Abstract

Vacuum cathodic arc TiN coatings with overlaying TiO_2_ film were deposited on polished and surface roughened by electron beam modification (EBM) Ti6Al4V alloy. The substrate microtopography consisted of long grooves formed by the liner scan of the electron beam with appropriate frequencies (500 (AR500) and 850 (AR850) Hz). EBM transformed the α + β Ti6Al4V mixed structure into a single α’-martensite phase. Тhe gradient TiN/TiO_2_ films deposited on mechanically polished (AR) and EBM (AR500 and AR850) alloys share the same surface chemistry and composition (almost stoichiometric TiN, anatase and rutile in different ratios) but exhibit different topographies (S_a_ equal to approximately 0.62, 1.73, and 1.08 μm, respectively) over areas of 50 × 50 μm. Although the nanohardness of the coatings on AR500 and AR850 alloy (approximately 10.45 and 9.02 GPa, respectively) was lower than that measured on the film deposited on AR alloy (about 13.05 GPa), the hybrid surface treatment offered improvement in critical adhesive loads, coefficient of friction, and wear-resistance of the surface. In phosphate buffer saline, all coated samples showed low corrosion potentials and passivation current densities, confirming their good corrosion protection. The coated EBM samples cultured with human osteoblast-like MG63 cells demonstrated increased cell attachment, viability, and bone mineralization activity especially for the AR500-coated alloy, compared to uncoated polished alloy. The results underline the synergetic effect between the sub-micron structure and composition of TiN/TiO_2_ coating and microarchitecture obtained by EBM.

## 1. Introduction

The surface characteristics of the implant material predetermine how the biomolecules absorb to the surface [1]. The adhesion proteins such as extracellular matrix proteins, cell membrane proteins, and cytoskeleton proteins influence bone tissue cells’ morphology, proliferation, and differentiation. The topography, chemistry, and surface energy also determine cell behavior in contact. The micro-textured implants demonstrate improved osseointegration (direct apposition of bone tissue to the implant) instead of smooth implants by in vivo test [2]. Therefore, the organization of surface roughness is an important parameter for the contact guidance phenomenon. The micron-roughened surface morphology can increase the surface area of the implant and the contact between the implant and host bone. In addition, it was demonstrated that osteoblast cells can detect and respond to different nano-topographical features in vitro [3]. When nanoscale architecture is combined with underlying rough microstructure, significant improvement in both implant fixation strength and matrix mineralization can be observed [4].

Among metallic implant materials, titanium and its alloys are considered to be the best choice for bone implant applications because of their low modulus of elasticity, corrosion resistance, and biocompatibility [4]. Unfortunately, Ti6Al4V alloy, which is most widely used, has low wear resistance and the debris generated during wear accelerates electrochemical reactions between the physiological environment and implant surface [5]. Moreover, the alloy loses biocompatibility if V and Al ions are released in human tissue, because Al can cause neurological diseases while V ions are cytotoxic [6]. Therefore, further developments that extend the longevity and bioactivity of these implant materials are needed.

For improving osteoconductivity and osteoinductivity of titanium implants, bioceramic coatings such as nanoclays [7], hydroxyapatite [8], and calcium silicate [9] have been utilized. However, these coatings demonstrate insufficient strength and fracture toughness for load-bearing applications in implantology [10]. TiN coatings applied to orthopedic alloys are well-known materials that positively affect the tribological properties of bone grafts by reducing the coefficient of friction [11], wear [12] and increasing hardness and scratch resistance [13]. Such tribological performance of a coating will help to enhance the service life of articulating implants if at high contact pressure the elastic recovery of the hard coating and soft alloy does not differ substantially. TiN-coated Ti6Al4V alloy also demonstrated biocompatibility [14] and reduction of ion release rate [15] compared to uncoated alloy. Nonetheless, studies of cell proliferation and differentiation on TiN-coated materials are contradictory: several papers report no difference in proliferation compared to controls [16,17], while other studies demonstrate an increase in proliferation [18]. Moreover, concerns were raised about PVD-coated Ti6Al4V with TiN because it suffered from coating failure due to either defects in the film [19] or higher difference in elastic modulus of Ti6Al4V substrate and TiN film [14].

On the one hand: this condition could be improved by applying suitable initial surface modification such as electron beam treatment to increase surface roughness and raise surface hardness values and, hence—elastic modulus of Ti6Al4V alloy before TiN deposition. That will reduce the risk of delamination due to the gradient-like hardness profile of the substrate. A feasible method for producing micro- and sub-micrometer surface texturing on metallic materials is the direct laser interference patterning (DLIP) that directly removes material by ablation [20]. However, when utilizing double-pulse generation 50% of laser energy is lost while treating metal surfaces [21] which makes the depth of the substrate strengthening lower. According to some authors [22], the formation of preferred crystallographic orientation that influences mechanical properties can explain the differences in the hardening mechanism of laser- and electron-beam processed metal surfaces. EBM can be carried out in different geometries of scanning (linear, circular, etc.) because charged electrons can be deflected and precisely guided [23]. This modification leads to direct structuring of the surface by periodical topography in a high vacuum state. Thus, low pressure prevents oxidation of easily oxidized titanium alloys and hinders the formation of brittle surface layer (the so-called alfa case) that affects negatively the mechanical properties and fatigue resistance of the alloy [24].

On the other hand, to increase bone formation and bonding strength, deposition of TiO_2_ coating on TiN film’s surface is a promising approach that could further improve the corrosion resistance of the coating [25], covering the nitride defects. TiO_2_ coatings are well-suited as protective non-toxic hydrophilic coatings in medical applications in which additional benefits like self-cleaning and anti-bacterial effects can be obtained [26]. It was also shown that anatase and mixtures of anatase-rutile crystallographic forms of TiO_2_ exhibited more efficient apatite formation than amorphous structures [27]. Additionally, nanostructured TiO_2_ exhibited higher bone growth values and bone-to-implant contact area [28]. Since the biological response is affected by the chemistry and topography of the implant material, the osteoblastic response of the cells to deposited TiO_2_ on the surface of TiN film is a highly interesting field of research.

A number of different techniques have been used for producing ceramic TiN and TiO_2_ films such as magnetron sputtering [29], high power impulse magnetron sputtering (HIPIMS) [30], CVD [31], ion beam-enhanced deposition [32], electron-beam deposition [33], low pressure plasma spray [34], dual plasma deposition [35], laser deposition [36], plasma ion nitriding [37], plasma immersion ion implantation [38], cathodic arc deposition [39,40,41]. The low process temperature allows the coating material to be deposited without reduction in hardness, corrosion resistance or distortion compared to techniques conducted at higher temperatures [42]. Except for low-temperature deposition, cathodic arc deposition offers an effective source of ionized, energetic material to produce well adherent and dense coatings at high deposition rates [43]. However, this technique has not been fully exploited for the production of biocompatible coatings on Ti6Al4V substrates. In our previous studies, TiN/TiO_2_ coatings were found to improve the hydrophilicity and anti-corrosion properties in Ringer solution of titanium alloy with lower aluminium content (Ti5Al4V) [44,45]. At the same time, we demonstrated that TiN/TiO_2_ coatings deposited on polished Ti6Al4V promoted cell adhesion without inhibiting cell viability [46].

The objective of this study was to create micro- and nanoscale architecture by a hybrid treatment approach that includes initial electron beam surface modification of Ti6Al4V substrate which increases its hardness and roughness, and subsequent deposition of TiN/TiO_2_ coatings. The dual-surface treated samples were characterized (composition, crystallinity, texture, surface topography) and their wear, corrosion resistance, and in vitro cytocompatibility using MG-63 osteoblastic cell line was evaluated and compared. To the best of our knowledge, the present study reveals for the first time that the proposed combination of surface treatment of the implant alloy displays adequate mechanical and in vitro biocompatibility, as well as corrosion protection. A systematic understanding of the factors that influence the behavior of the implant systems provides the basis for further investigation into the production of coated biomedical devices with controllable performance.

## 2. Experimental Methods

### 2.1. Substrate Treatment and Coating Deposition

Samples with a chemical composition of 6.22% Al, 3.57% V, 90.38% Ti (in wt%) and dimensions Ø22 × 4 mm were cut by using the electro-erosion cutting method from round Ti6Al4V alloy. The surfaces of the samples were mechanically grounded, polished, and ultrasonically cleaned in water. Electron beam modification (EBM) was carried out by electron beam installation Leybold Heraus (EWS 300/15–60). The following technological parameters were applied: linear manner of scanning, electron beam current—I = 30 mA, accelerated voltage—U = 52 kV, speed of the sample’s motion—ν from 0.5 up to 2.5 cm/s, electron beam frequency—f = 500 up to 1000 Hz. After EBM, the disks were rinsed in distilled water, ultrasonically cleaned in ethanol, acetone, isopropanol, and water and dried in a stream of air. The deposition of TiN (by vacuum cathodic arc technology at 340 °C) and overlaying of TiO_2_ (by glow plasma discharge at 300 °C) have been described in detail elsewhere [44]. In brief, the samples were attached to a holder that rotated at 0.5 Hz during deposition. The samples were bombarded by an arc-enhanced glow discharge for 5 min. to remove any oxides and trace contamination. The deposition was carried out in a flow of pure N_2_ at working pressure 7.5 × 10^−1^ Pa and substrate bias 250 V. The substrate temperature in a vacuum was monitored by a thermocouple. A sputtering system located in the same chamber was used for the deposition of TiO_2_ in a pure O_2_ atmosphere at a working pressure of 6 × 10^0^ Pa for 240 min. A summary of the deposition parameters is shown in Table 1.

### 2.2. Characterization Techniques

The phase composition of the substrates and deposited coatings were examined by X-ray diffraction (XRD, URD-6 Seiferd&Co diffractometer) operating with CuKα radiation (λ = 0.154178 nm) in a symmetrical Bragg–Brentano mode. A step size of 0.1° and counting time of 10 s per step were applied. The texture coefficient (TC) for each (hkl) reflection was calculated after subtraction of background radiation according to the equation in [47]. The weight fractions of rutile (W_r_) and anatase (W_a_) phases in the coatings were determined following the procedure described in [48], while the grain size (D) was determined by the Debye–Scherrer equation. Dislocation density (δ) which represents the number of defects in the films was calculated by using the equation shown in [49].

Further details are given in Supplementary S2.1. Scanning electron microscopy (SEM, LYRA I XMU, Tescan, Brno, Czechia) at an energy of 10 and 20 kV was used to characterize the surface morphology, coatings, and cross-section cuts of the substrates. The surface topography was analyzed by using a contact profilometer (Mitutoyo SJ 201 P, Kawasaki, Japan). The samples were evaluated quantitatively for R_a_ (average roughness) and R_z_ (maximum roughness height) values after ten times scanning with a cut-off length of 0.8 mm and 5 sampling lengths (according to ISO 4288:1996) in a direction perpendicular to grooves caused by local melting. The surface nanotopology was characterized by atomic force microscopy (commercial AFM system Q-Scope™ 250/400 Nomad™ (Ambios Technology, Inc., Santa Cruz, CA, USA). Measurements were taken by using a 10 nm radius silicon tip on each coating at 50 × 50 μm scan areas. The depth profiles of Ti, N, and O were measured by a glow discharge optical emission spectroscopy (GDOES, GDS-750 QDP, LECO Instruments, St. Joseph, MI, USA) under conventional plasma conditions [50].

Micro-Vickers hardness measurements were made on the top of each substrate (polished and EBM) with 200 g load and dwell time of 15 s by using microhardness ПМТ-3 (ПОМО) tester. The distance between the indents was 20 μm. To compare the in-depth microhardness changes after EBM, cross-sections of AR500 and AR800 were made and measured with a load of 100 g. The nanohardnness of coatings was investigated by employing a nanomechanical tester (Bruker, Billerica MA) applying a load of 25 mN with a Berkovich indenter. During the test, 48 indentations with a spacing of 80 μm were made. The adhesion of coatings was characterized by using a CSEM-Scratch tester with a standard Rockwell-C diamond indenter (up to a load of 50 N) and by applying optical microscope observations. The coefficient of friction (COF) was determined by a sliding wear study performed at a ball-on-flat tester (UMT-2M (Bruker-CETR) tribometer (normal load 2 N, linear speed 100 mm/s, time of testing 300 s) with a sliding Ø6 mm ball coated with Cr. The sliding rate and time were chosen to initiate the wear of the coatings. The tests were conducted in air and at room temperature.

The electrochemical tests were performed in a three-electrode corrosion cell comprised of a platinum counter electrode, a saturated Ag/AgCl (E = 210 mV vs. SHE) reference electrode, and a working electrode immersed vertically. The area of the working electrode exposed to the solution was equal to 0.2 cm^2^. All electrodes were immersed in 80 mL naturally aerated phosphate buffer saline (PBS) containing 8 g/L NaCl, 0.2 g/L KCl, 1.44 g/L Na_2_HPO_4_, 0.24 KH_2_PO_4_ (Sigma Aldrich, Hamburg, Germany) with pH 7.4 maintained at body temperature (37° ± 0.5 °C) by a thermostat. The electrochemical measurements were carried out by using a potentiostat/galvanostat 263A (EG&G Princeton Applied Research, Oak Ridge, TN, USA) coupled with a PC by a controller. Before the measurements, the samples were allowed to stabilize under open circuit potential (OCP) for 30 min. After stabilization, potentiodynamic polarization curves started at a potential of about −250 mV vs. OPC up to +2000 mV vs. Ag/AgCl using 1 mV.s^−1^ potential scan rate. The corrosion parameters E_corr_ (corrosion potential) and j_corr_ (corrosion current density) were extracted from Tafel plots while polarization resistance (R_p_) was determined from the Stern–Geary equation. All parameters were determined by using PARCalc Tafel Analysis software.

### 2.3. In Vitro Cell Culturing

The influence of AR surface modification of the samples on cell attachment efficiency and growth was assessed with human osteosarcoma cells (MG63, CRL-1427). The cells were maintained in Dulbecco’s Modified Eagle Medium (DMEM, Gibco, Waltham, MA, USA) containing 10% fetal bovine serum (FBS, Lonza, Basel, Switzerland), 100 units/mL penicillin, and 100 µg/mL streptomycin in a humidified CO_2_ atmosphere at 37 °C. They were routinely checked for mycoplasma contamination by 4,6-Diamidin-2-phenylindol staining (DAPI, Sigma-Aldrich, Hamburg, Germany) and were found free of it. The cells were seeded with a density of 4 × 10^3^ cells/cm^2^ in complete DMEM media on the sample surface in 6-well plates. Six hours later, after 3 washes with phosphate-buffered saline (PBS), the cells were detached with 1 mL 0.25% (*w*/*v*) Trypsin—EDTA (Lonza) for 5 min, centrifuged at 1000× rpm for 5 min, and used for cell counting with Countess™ Automated Cell Counter (Invitrogen, Waltham, MA, USA) to determine the cell attachment. The cell attachment efficiency was defined as (number of attached cells/number of plated cells) × (100%) and expressed as mean ± standard deviations.

The cell growth was determined by MTT assays [51]. Briefly, an MTT solution (5 mg/mL) was added to each well, where MG63 cells were grown for 24 h on 3 cm^2^ AR or EBM surfaces in 3 mL media and were further incubated for 3 h at 37 °C. To dissolve the formazan product of the MTT, cell media was removed and 300 µL/surface of 100% anhydrous isopropanol was added. Following complete extraction of formazan, the samples were removed and the optical density of the obtained solutions was measured at 550 nm with a DTX880 spectrophotometer (Beckman Coulter, Brea, CA, USA). The results were used to calculate cell growth by the OriginLab program.

Immunofluorescence was used to observe cell morphology and cytoskeletal distribution.

The cells were seeded with a density of 1 × 10^4^ cells/cm^2^ in complete DMEM media on the sample surface in 6-well plates. Twenty-four hours later, after three washes with PBS, they were fixed with 4% formaldehyde solution for 20 min. The cell morphology was detected by F-actin staining with AlexaFluor-568 Phalloidin (Invitrogen, USA). Following three washes with PBS and two in water, the slides were mounted in UltraCruz fluorescence mounting medium (Santa Cruz Biotechnology, Dallas, TX, USA). Fluorescence microscopy was performed with Carl Zeiss Axiovert 200M Inverted Microscope.

IRDye 800CW BoneTag Optical Probe (Li-Cor) was used to detect the ongoing mineralization process in MG63 cells on AR and EBM surfaces. IRDye 800CW dye has an extended fluorescence signal detection to the near-infrared fluorescent region of the spectrum without affecting the ability of the compound to function as a marker of the mineralization process. NIR fluorescence detection improves the penetration depth due to low cell autofluorescence, translating to low background interference. Following 31 days of growth of MG63 cells on AR or EBM surfaces, the cells were incubated for 24 h with 2 nnol/3 mL IRDye 800CW and fixed with 4% formaldehyde solution for 20 min. The samples were washed with PBS containing 0.02% Triton X-100 to remove the unbound dye. In-Cell Western Assay was used for normalization by a CellTag™ 700 stainings. The fluorescence was detected with Odyssey infrared imaging system equipped with an application software v.3 (Li-Cor).

The data were evaluated by analysis of variance (ANOVA) followed by Bonferroni’s post-hock test. Differences in results at the level of *p* < 0.05 were considered statistically significant. The statistical analysis was carried out by using PASW 18.0 statistical software package (IBM) for Windows. All experiments were performed in triplicate.

## 3. Results and Discussions

### 3.1. Preliminary Experiments

To obtain increased surface hardness and roughness, the speed of sample motion (ν) and scanning frequency (f) of the electron beam were changed while the electron beam current was kept constant (30 mA). The change in hardness and roughness of substrate before and after EBM is shown in Table 2.

The speed of sample motion (ν) refers to the heating and cooling rate of the treated surface. The higher the speed of sample motion, the lower the heat input. Both the speed of sample motion and beam power determine the width, thickness, and volume of the treated area. When changing the speed of sample motion (ν) and keeping scanning frequency (f) equal to 1 kHz, the highest hardness was obtained at 2 cm/s. At that speed of sample motion, the cooling rate allows the formation of the highest amount of martensite. However, the surface hardness was exceptionally high and the surface of Ti6Al4V alloy was cracked perpendicularly to the linear trajectory of the moving electron beam. Moreover, the measured surface roughness was comparatively low (R_a_ 1.07–1.20 μm). To increase roughness values while keeping surface hardness high without cracking, the scanning frequency (f) was varied from 500 up to 850 Hz. As seen in Table 1, the highest hardness and roughness values were obtained for scanning frequency of 500 Hz (indicated from now on as AR500). With the increase in scanning frequency, the roughness values decreased, while the hardness values kept almost the same. Because of the small difference in R_a_ values for AR500 and Ti6Al4V modified at a scanning frequency of 650 and 750 Hz, the other samples chosen for the next examinations were those treated with f = 850 Hz (indicated as AR850). A schematic view and codification of the samples are shown in Figure S1.

### 3.2. Structure, Hardness, and Roughness of the Substrates

**Figure 1:**
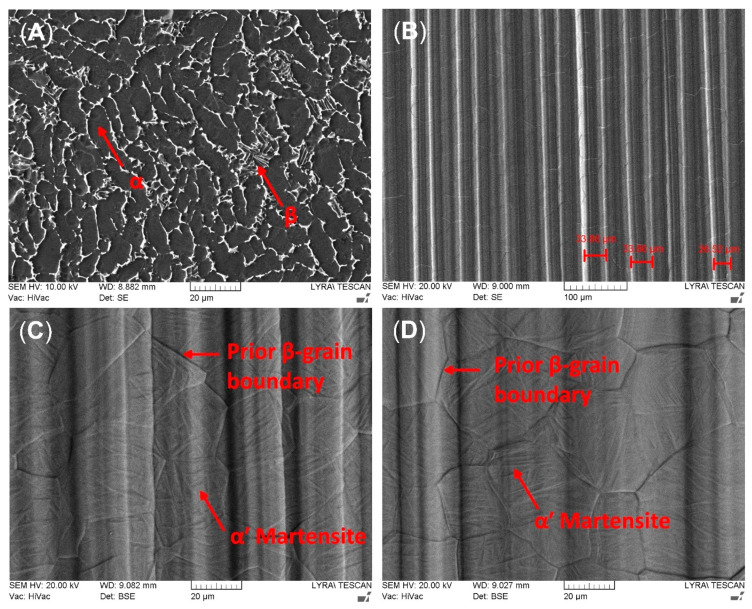
Representative SEM micrographs of (**A**) the initial structure of Ti6Al4V alloy, (**B**) EBM surface of AR500 substrate; (**C**,**D**) top surface microstructure of EBM AR500 and AR850, respectively, seen at higher magnification.

Figure 1A shows the initial microstructure of Ti6Al4V alloy. It consists of lamellar α-grains with an intergranular β phase. Figure 1B displays the orientated structure with deep grooves on the surface of titanium alloy after EBM. SEM measurements of densities of irregularities on the alloy’s surface are shown in Figure 1B. The average distance between the grooves was found to be 42.67 ± 1.58 μm and 32.34 ± 0.82 μm for the AR850 and AR500 samples, respectively. Nonetheless, the average maximum peak-to-valley (R_z_) values were equal to 5.84 ± 0.17 μm and 7.35 ± 0.21 μm, respectively. At higher magnification (Figure 1C,D), it could be seen that within the prior β grains, the microstructure consists of individual-oriented martensite laths with a width of above 1–2 μm, which are expected for the diffusionless transformed β-grains under rapid solidification conditions typical for EBM. The inclination angles in the obtained anisotropic structure follow Burger’s relation between prior β and α’ phases [52]. The lack of α-phase grains along the prior-β grain boundaries suggests that (1) the cooling rate after EBM is higher than 410 °C/s (the critical cooling rate for the displacive transformation of Ti6Al4V) [53] and (2) high thermal gradient (10^4^–10^5^ °C/cm) in the re-melted area [54]. The thermal effect of electron beam irradiation causes a surface with a large grain size after rapid re-solidification. The equiaxed β phase formation during heating is considered to be driven by reducing interfacial energy [55].

**Figure 2:**
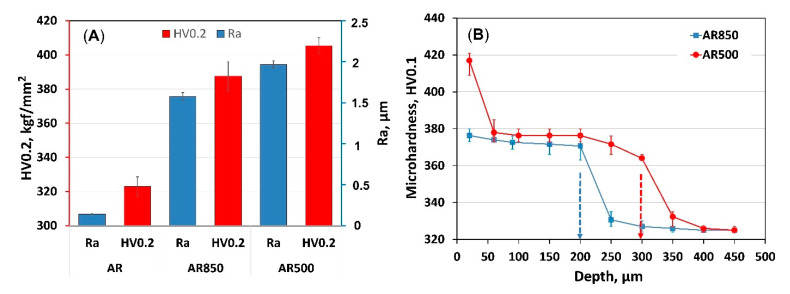
Comparison of (**A**) roughness values and surface hardness, and (**B**) cross-section hardness of EBM substrate material.

The hardness and surface roughness values plotted in Figure 2A are arithmetic mean values of 10 measurements per specimen with error bars representing ± standard error. The roughness and hardness results show that they increased significantly after EBM. The initial hardness of the alloy was 323 ± 5.6 HV0.2. Similar hardness values for the AR alloy were reported by [56], while Young’s modulus varied between 116–120 GPa [57]. The highest surface roughness (1.97 μm) and average hardness (405.4 HV0.2) were measured for AR500 samples followed by AR850 where the hardness was almost 387.5 HV0.2 whereas the Ra was equal to 1.58 μm. Similarly, nanoindentation measurements revealed an increase in hardness of electron beam additive manufactured Ti6Al4V with α’-martensite structure equal to 6.5 GPa while Young’s modulus did not change substantially (128 GPa) [58].

Because of the effect of electron beam bombardment, the microhardness profiles in depth (Figure 2B) exhibit a steady decrease with an increase in distance from the surface. That effect could be attributed to thermal stresses and martensite transformation during rapid cooling and re-solidification. The micro hardening cross-section depth is more profound for the AR500 substrate (around 300 μm) than the AR850 alloy (about 200 μm). Because of variation in the cooling rate with scan frequency, the depth of α’-martensite transformation decreases.

### 3.3. Structure, Roughness, and Composition of the Coatings 

**Figure 3:**
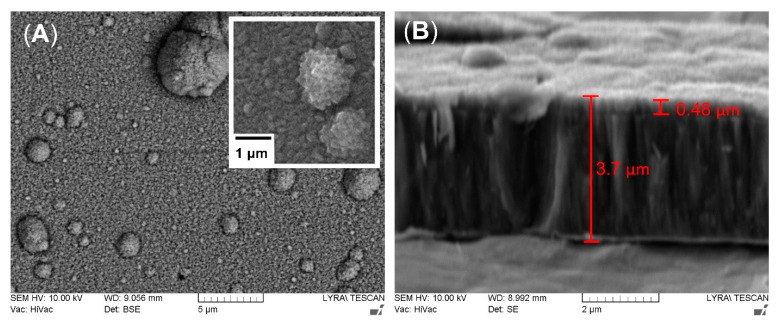
Representative SEM micrographs of the top view (**A**) and cross-section (**B**) of TiN/TiO_2_ coating. Enlarged magnification reveals the surface morphology of the oxide crystals. The measurement of whole thickness of the coating includes the white-looking intermediate pure titanium layer below TiN sublayer.

The surface structure of the deposited TiN/TiO_2_ coating (Figure 3A) is composed of islands (microparticles) protruding from the nitride sub-layer and covered with oxide crystals. Those large conglomerates of atoms are typical for cathodic arc deposited films and are thought to be the main drawback of this technique. They are formed during cathodic arc evaporation because of intense localized heating of the titanium target. As seen in Figure 3A, the microparticles’ size is not constant but droplets are homogeneously distributed in and over the coating. The droplet phase is characterized by its conical morphology. However, the resulting rougher morphology of the film could be beneficial for cell-implant interactions. It should also be considered that shallow potholes around these droplets in the film are possible [59]. However, the higher the applied substrate bias, the denser the coating. The cross-section structure revealed long and fine nitride columns extending from the substrate and thinner (about 0.48 μm) overlaying oxide layer (Figure 3B). The nitride layer morphology is predetermined by the high energy of the ionized particles. While the negative substrate bias repels the electrons, the energized ions are attracted by the negatively charged substrate with energy enough to sputter the surface atoms and diffuse to sites with lower energy that produce higher density films. Moreover, with the increase of the applied bias, the film becomes rougher because of the re-sputtering effect, which results from the competition between ion bombardments of the coating and the etching of the evolving film [60].

**Figure 4:**
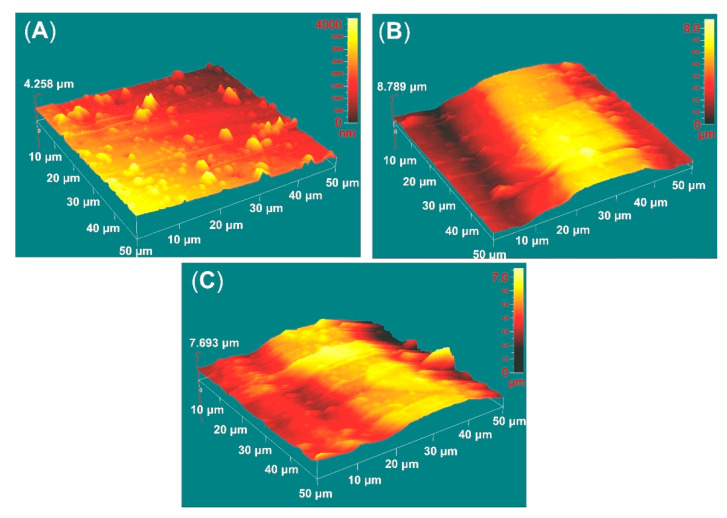
AFM deflection images of TiN/TiO_2_ coatings deposited on the polished AR (**A**), AR500 (**B**), and AR850 (**C**) samples.

Figure 4 shows the AFM deflection images of the films deposited on AR and EBM samples. The average roughness data of the deposited films were obtained over an area of 50 × 50 μm and are presented in Table 3. The roughness values tend to increase after EBM of the substrate since the coating replicates the substrate morphology. The mean roughness (S_a_) is over 2 times higher for the EBM-coated sample. In contrast to the previously measured R_a_ values by contact profiler, S_a_ of the coated AR sample is increased because of the presence of droplet phase, whereas the roughness values of EBM samples after coating did not change substantially (see Table 2 and Table 3) because of the pre-existing uneven morphology of the martensite laths (Figure 1C,D). Due to squaring the amplitude in its calculations, S_q_ (root mean square roughness) can be considered more sensitive to valleys and peaks than S_a_. The vertical distance between the highest and the lowest points in the evaluated area (R_pv_) is also considered important because it contains information about the extreme features of the surface. The skewness (S_sk_) indicates heights’ distribution asymmetry, which in the case of complete symmetry is equal to zero. As seen in Figure 4A, the higher than zero AR sample values demonstrate that the heights are larger than the valleys, while those of AR500 samples are almost symmetrically distributed. For AR850, the valley areas predominate.

**Figure 5:**
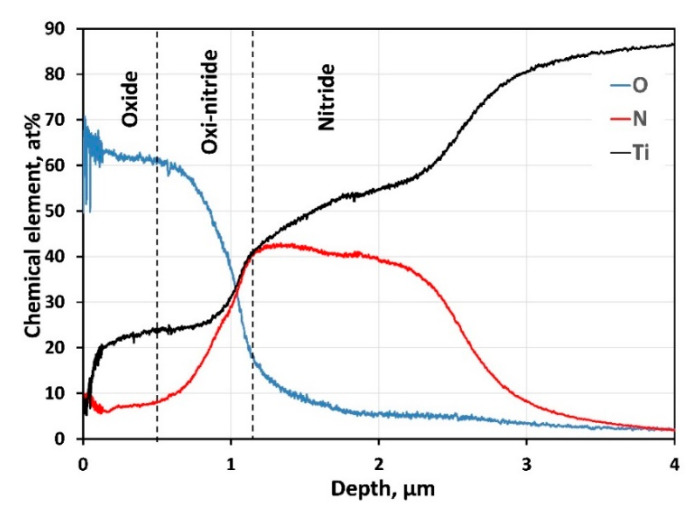
GDOES composition profile of the chemical elements in-depth of the coating.

Elemental profiles (Figure 5) obtained for Ti, N, and O clearly show titanium oxide’s presence in the coating’s outer surface. The element profile shapes in the oxi-nitride intermediate layer confirm the diffusion between both layers. Although deposited at high nitrogen pressure, the increased flux of Ti target atoms to the substrate causes slightly reduced nitrogen content in the nitride film instead of the stoichiometric ratio. The atomic concentration of N gradually decreases in-depth, reaching the substrate.

### 3.4. Phase Composition

**Figure 6:**
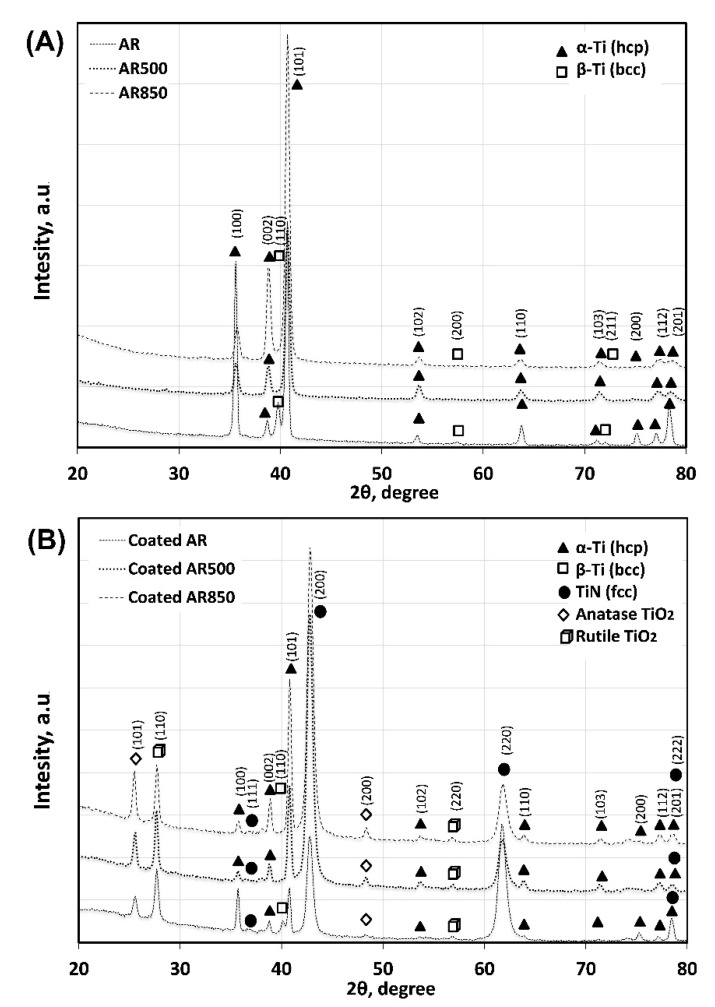
XRD spectra of the substrates (**A**) and deposited coating (**B**).

Figure 6 shows typical XRD spectra from the substrates and coatings. After EBM, the peaks corresponding to the β phase (JCPDS No. 09-0098, space group Im-3m) disappear because of the transformation of a large portion of β to α phase (JCPDS No. 01-1198, space group P63/mmc) near the surface. The decrease in (100), (110), (200), and (201) planes together with the increase in the intensity of (002), (101), and (102) planes of α-Ti indicate re-orientation in the micro-volumes of the α-phase grains and a change in texture (Table S1, Figure 6) after EBM. This re-orientation affects the texturing of overlying TiN (JCPDS No. 87-0633, space group Fm-3m) that changes from (220) on the AR substrate to strong (200) orientation when TiN was deposited on EBM substrates (Table S1). The average grain sizes of α-Ti, TiN, and TiO_2_ calculated by using Scherrer’s equation are shown in Table S2. Their values for all coated samples are close because the films are grown under the same experimental conditions.

Few reports in the literature discuss the contribution of near-surface substrate crystallography to TiN film’s orientation subsequently grown on them [61]. According to Li et al., crystallographic growth is preferable to that plane that has the best elastic match with the substrate [62]. On the one hand, TiN (200) and basal Ti (002) planes are close-packed while TiN (220) and prismatic Ti (100) planes are less dense. On the other hand, the application of substrate electrical bias during nitride deposition allows the attraction of charged ions with high kinetic energy which increases condensation energy. The higher the substrate bias, the higher the substrate temperature at which diffusion occurs more rapidly, and recrystallization may occur, resulting in higher density films with larger columnar grains. Compared to AR Ti6Al4V alloy, α’-martensite is a metastable phase that contains a high density of dislocations (Table S2) and twins. Such a structure will decrease the free mean free path of phonon triggering a reduction in substrate thermal conductivity and its surface temperature will be higher. It was reported that higher temperature deposition results in predominant growth with a fully dense (200) orientation on the initial monolayer of the film [63]. It is commonly accepted that (200) texture can be formed when high energetic bombardment is applied resulting in re-nucleation during coating growth [64]. Similar results indicating that EBM of the substrate is beneficial for the preferred (200) orientation were confirmed in our previous studies on magnetron sputtered TiN/TiO_2_ coating deposited on EBM Co-Cr alloy [65]. This (200) orientation with a low density of atomic packaging allows “channeling” that transfers the energy of particles into the surrounding of these channels [66].

In contrast, (220) texture is still not well theoretically understood. The (220) orientation was proposed to be present when N atoms form tetrahedral dumb-bell pair along (220) direction with NaCl lattice [67]. Since the stopping energy of coatings is proportional to the distance of ions must transfer, (220) plane has lower stopping energy (Figure S2) because ions should travel greater distances to distribute energy. In other terms, there is more open space between atoms for impinging species to dissipate energy without re-sputtering. The stopping energy is important when an excessive ion bombardment process such as in a cathodic arc with high applied substrate bias is present. The overall energy of the system is reduced by re-sputtering (200) facets resulting in an increased fraction of (220) [68,69], which has a lower re-sputtering rate.

**Figure 7:**
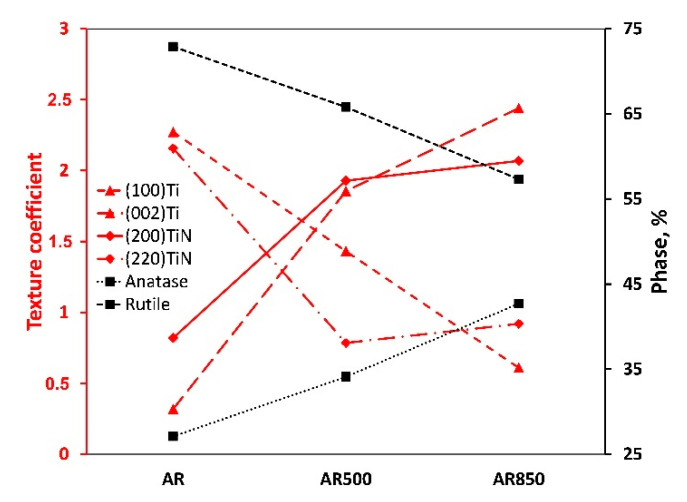
Texture coefficients of (100)Ti, (002)Ti, (200)TiN, (220)TiN, anatase, and rutile phase percentage in AR, AR500, and AR850.

As indicated in Figure 7 and Table S1, oxidation of differently textured TiN leads to the formation of crystalline TiO_2_ phase with a different rutile-to-anatase ratio. During the glow plasma discharge deposition of oxide, the energy of particles is enough to cause predominant rutile (TiO_2_, JCPDS No. 21-1276, space group P4_2/mnm) and a smaller amount of anatase (TiO_2_, JCPDS No. 89-4921, space group I4_1/amd). Generally, most titanium oxide films deposited by magnetron sputtering are amorphous or consist only of the anatase phase [70]. Higher temperatures will increase the probability of TiO_2_ crystallization. Since the rutile phase needs more energy to be synthesized it proves the oxide film’s energetic condensation. However, A. Seifitokaldani et al. [71] used a computational approach to study oxygen adsorption of two major facets of TiN, namely (111) and (200), and found strong oxygen absorption on TiN(111) while keeping the molecules on its surface. In contrast, TiN(200) showed a very feeble absorption bond and the ability of oxygen to take a significant distance from the surface, thus providing new sites for the next oxygen molecule adsorption. Moreover, it is known that crystal orientation can influence the thermal conductivity of vacuum-deposited films [72]. Then, both the packaging factor of the film and different oxygen adsorption of the major facets make so that the increase in (200)TiN intensity and the decrease in (220)TiN plane intensity contribute to the formation of higher amounts of less stable anatase phase. The slight enlargement in oxide crystallite size on EBM samples may be interpreted as a result of increased adatom mobility triggered by the crystalline orientation of nitride. On the other hand, although the columnar structure of nitride facilitates conductivity [73], the thermal conductivity of nitride film is reduced when increasing the concentration of boundaries, dislocations, and other defects. As shown in Table S1, the dislocation density of TiN nitride on the AR sample was found to be higher than those of EBM and coated specimens. That fact also contributes to a higher rutile-to-anatase ratio.

### 3.5. Mechanical and Tribological Properties

Since the whole coating thickness is equal to 3.7 μm, the penetration depth at 25N applied load does not exceed 1/10th of its thickness. Therefore, substrate hardness does not affect the measured nanohardness value of the coatings. However, the measured values of the coatings’ nanohardness (Figure S3, Table 4) represent the mid-term value of oxide and nitride layers. A film’s hardness is correlated to its density, phase composition, residual stress, and grain size. The film’s density depends on the energy of particles reaching the surface of the growing film, which is similar for all coated samples. The observed higher hardness of the coating on AR alloy, on the one hand, could be attributed to the smaller size of nitride and oxide crystal grains of the AR sample, while coatings on EBM substrates had relatively larger grains (Table S2). On the other hand, texture orientation changes from (220) in AR-coated sample to (200) within EBM samples. The higher hardness of (220) textured TiN film as opposed to (200) orientated Chawla et al. attributed to the less active slip systems of the former [74]. Moreover, the hardness value drop could also be due to the higher surface roughness values [75] because the flat sample exhibits better surface properties. Not only TiN, but also ТiО_2_ film is responsible for the difference in hardness values measured. Kaczmarek et al. examined magnetron sputtered anatase coating with a crystalline size of 33 nm that displayed a hardness of about 3.5 GPa (Young modulus 115 GPa), whereas rutile film with 74 nm-sized grains showed hardness equal to 7.9 GPa (elastic modulus 138.5 GPa) [76]. It follows that the increased anatase fraction on the surface of the EBM sample also contributes to the decreased nanohardness value as opposed to that of the coated AR alloy that contains the highest amount of rutile phase. All those findings are completely in line with the results obtained in the present study.

In general, materials with higher hardness show better wear resistance. The H^3^/E^2^ ratio represents the plastic deformation resistance of coating related to the contact behavior of the films [77]. The higher values indicate the ability of a coating to absorb more energy (Table 4). The calculations suggest that that behavior should correspond to the increase in hardness values, and the coating on the polished substrate should be more rigid to contact compared with the others.

**Figure 8:**
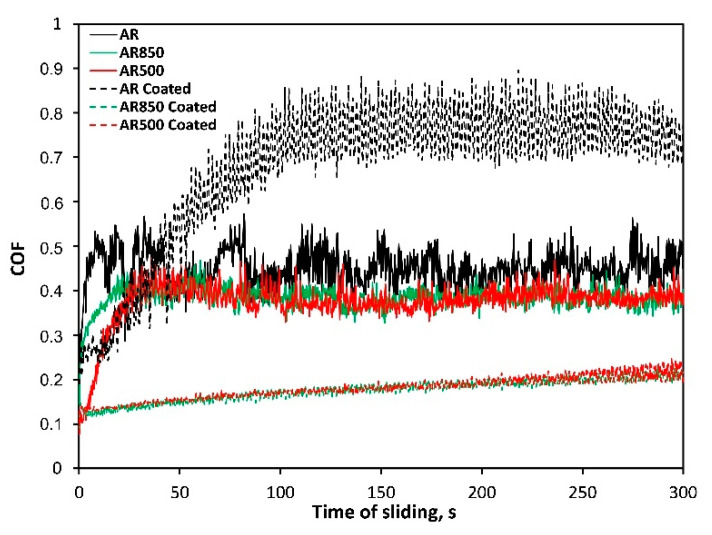
Representative friction curves of bare and coated samples in un-lubricated sliding against ball coated with Cr.

The surface roughness and hardness are among the parameters that determine the quantity of friction. COF of the bare AR substrate fluctuates significantly, whereas those of EBM uncoated samples remained around 0.37–0.38 at a relatively steady stage (Figure 8). The reduction of COF is due to the increased surface hardness of the modified substrates unlike those of AR Ti6Al4V alloy. The wear profiles of rubbing ball surfaces indicate the lowest COF for EBM and coated samples compared to other contacting pairs. The wear resistance of coatings was compared by estimating the width of the wear track. Because of the ductile substrate, the severe friction between coated AR specimen and the ball turned from sliding into plowing [78] and greater surface damages due to the plastic deformation of the substrate (see Table 5, wear track width) were observed. The wear scar was about 552.4 μm in width. After a certain period, because of the interaction between the hard coating and ball material, abrasive wear took place that accelerated the wear of coating on the ball head as the wear test progressed. 

In principle, roughеr surface experiences greater asperity contact increasing the tendency to plastic deformation during sliding and formation of wear debris after a certain sliding distance. However, rougher coated EBM surfaces demonstrate the lowest COF and highest wear resistance because of low adhesion between contact surfaces and smaller contact pressure generated on the EBM surface of TiN/TiO_2_ coatings. The wear scar widths were equal to approximately 215.5 and 216.5 μm for the coated AR500 and AR850 samples, respectively (Table 5). There were no significant differences in COF of the coated EBM substates which suggests that deposited film is durable and protective on both modified alloys.

**Figure 9:**
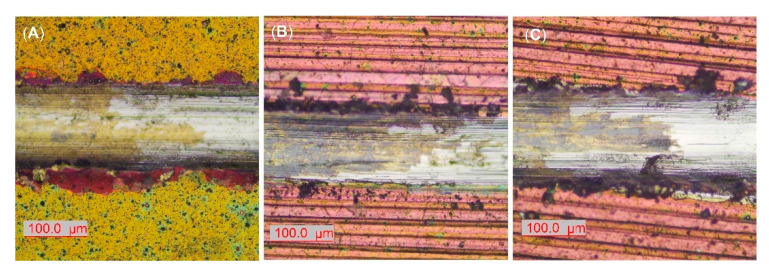
Representative micrographs of scratch tracks at adhesive critical loads (Lc) of (**A**) АR; (**B**) AR850; (**C**) AR500 samples.

It is known that adhesion strength between coating and substrate is a critical property in the wear resistance of thin films. During the dynamic scratch testing, the critical adhesive loads (Lc) indicate resistance to coatings’ delamination. The interfacial resistance of the polished and coated sample is lower as opposed to EBM samples (Table 5). That fact is also confirmed by optical micrographs of scratch tracks at critical loads where more severe lateral spallations were formed at the border of the AR sample (Figure 9A). The cracking of the coating is associated with the indenter penetration, higher film hardness, and plastic deformation of the substrate. The examined coating demonstrated higher adhesion strength on AR substrate in comparison to similar arc PVD deposited TiN coatings on polished Ti6Al4V [11,79] and Ti20Nb13Zr alloys [41] tested at similar conditions. Both coatings on EBM substrates show higher critical loads and, therefore, higher levels of adhesion force (Table 5, Figure 9B,C). Since the critical force in the scratch test depends on many factors including the hardness of the substrate [80], the increase in hardness of substrate by EBM had a beneficial effect on improving the adhesion strength of the coating. It could also be concluded that higher COF due to wear is related to weaker adhesion strength which accelerates the wear of the film under conditions of dry sliding. Since many factors affect coating wear, the coated samples’ tribological tests showed a lack of correlation between the H^3^/E^2^ ratio and COF values or wear resistance. It follows that the coating with higher resistance to plastic deformation becomes stiff with little susceptibility to plastic deformation. A lack of such correlation was also noted in [81].

### 3.6. Electrochemical Performance

**Figure 10:**
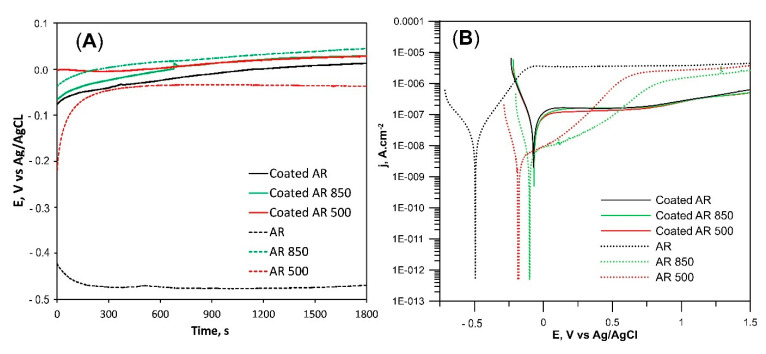
Representative open-circuit potentials (**A**) and potentiodynamic (PD) polarization curves (**B**) of the samples immersed in PBS at 37 °C.

Figure 10A presents the open circuit potential (OCP) of all bare and coated samples. OCP kept shifting positively during immersion in PBS for all samples except for AR specimens. That positive shift is related to the generation or presence of passive film, and, therefore, the anodic process is the dominant electrode process. The OPC of AR was maintained at approximately −0.47 V vs. Ag/AgCl after 30 min of immersion in PBS which is much lower than that of EBM and coated samples.

Figure 10B compares the potentiodynamic polarization curves of the samples immersed in PBS solution at 37 °C. The corresponding electrochemical parameters determined from polarization curves are given in Table 6. The similarities in cathodic branches of PD curves demonstrate that the main cathodic reaction in all specimens is the reduction of oxygen. The wide passive region indicates that the passive film is pitting-resistant and stable. The shape of the PD curve changed after the EBM of the substrate. j_corr_ values were smaller while E_corr_ values were more positive. Additionally, the current density values of EBM Ti6Al4V in the range 0–0.5 V were lower as opposed to the AR sample. All these changes indicate better corrosion resistance compared to AR alloy. On the one hand, those facts can be explained with differences in the microstructure of substrates. According to H.P. Hack, the different chemical composition of α and β phases in the AR sample can induce a galvanic effect and decrease the corrosion resistance [82]. Longhitano et al. found that the homogeneous distribution of both V and Al in one (α’) solid solution reduces the galvanic effect and is beneficial for corrosion resistance [83]. Moreover, some researchers considered that the presence of all elements in one phase stimulates the formation of a more stable oxide film as opposed to an AR alloy [84]. The lower j_pass_ values of EBM substrates indicate that the oxide film on the alloy surface is more stable and intact. Additionally, the decrease in grain size after EBM (Table S1), was also proposed to enhance the surface passivation properties [85]. On the other hand, the roughness and texture of EBM substrate differ from those of AR alloy. The area of the exposed surface to the solution plays an important role in determining the corrosion rate. The generation of the rougher surface by EBM increases surface area. Since the current is proportional to the actual surface area, it follows that with increasing real surface area on the rougher surface (although the real surface area of EBM samples was not quantified), current density decreases. Moreover, the untreated alloy demonstrates mainly prismatic (100) and (201) orientations whereas the electron beam irradiation textures the surface not only in (100) but also in basal (002), pyramidal (101), (102), (201), and other facets. Therefore, EBM samples have weaker texture exposing different crystallographic planes to the solution. In that connection, Martin et al. concluded that when various planes are exposed to corrosion, the weaker texture does not change corrosion behavior significantly as opposed to stronger textured Ti6Al4V alloy [86].

The almost identical PD curves of coated samples (Figure 10) indicate that their corrosion resistance is a function of the thickness and density of the coatings. The corrosion current densities for all coated samples were found to be higher than those of the bare substrates indicating a higher dissolution than the substrate alloy. That fact can be associated with the presence of micro defects in the film that could impact the electrochemical behavior. However, the current density at E > 0 V slowly increased and retained small values in the 10^−7^ A cm^−2^ range indicating very low dissolution. There were no oscillations in the passive region’s current density, which can be correlated to a lack of metastable pit formation. Simultaneously, the lowest j_pass_ values (Table 6) confirm the presence of intact and stable oxide on the surface of the film whereas E_corr_ values were the most positive measured among all tested samples. It can be said that the coatings exhibit high resistance to corrosion initiation than bare Ti6Al4V alloy. It therefore follows that the high thickness, structure, and chemical composition of coatings are responsible for film stability and its protection against corrosion.

### 3.7. In Vitro Biocompatibility

**Figure 11:**
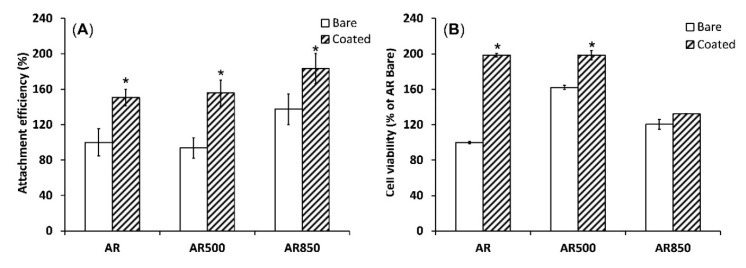
Cell attachment after 1 h (**A**) and cell viability evaluated by MTT assay (**B**) of MG63 growing on uncoated and coated samples for 24 h. * *p* < 0.01 compared to the AR sample, n = 2.

The supportive effect of TiN/TiO_2_ coatings on cell adhesion and viability was demonstrated when the polished AR sample was compared to the polished and EBM-coated samples. Compared to the uncoated group, TiN/TiO_2_-coated samples’ cell attachment efficiency was better (Figure 11A). It is known that nano- and submicron-structured crystalline particles have higher surface charge densities than bulk materials [87]. Therefore, the small crystalline TiO_2_ has a higher negative surface charge that is expected to show a high affinity to some positively/negatively charged cell-adhesion mediated molecules. Considering different extracellular matrix (ECM) proteins, Price et al. stated that due to its relatively linear and small molecule, vitronectin recognized by osteoblasts is preferentially adsorbed to nano-crystalline surfaces [88]. Moreover, cell attachment is affected by the surface roughness of samples. As opposed to AR and AR500 samples, AR850 specimens before and after coating deposition demonstrated higher attachment efficiency. Therefore, the surface’s organization in isotropic roughness with a higher distance between the grooves (above 42 μm), lower peak-to-valley (R_z_) values and predominate valley areas influence the attachment response of MG63 cells. It follows that cell adhesion can be governed by well-defined roughness features comparative to osteoblast cell size (approximately 20 μm [89]). That fact can be explained by the flexible and elastic external cell membrane that is prone to accommodate small features and/or complex surface topographies [90]. That initial accommodation can be better achieved on a less rough surface such as that on AR850 rather than on AR or AR500 irrespective of the presence/absence of the coating. Moreover, the change in surface energy related to coating structuring and phase composition was found to be able to modulate protein adsorption which further regulates cell adhesion and spreading [91].

The deposition of TiN/TiO_2_ coating also promoted cell viability during the initial 24 h compared to uncoated Ti5Al4V substrates (Figure 11B). However, cell viability on AR850-coated and the uncoated alloy was found to be lower than that on AR500 samples. It was claimed that micro- and nanometer peaks and valleys on implant surfaces were found to affect the cytoskeleton organization and intracellular transduction signaling pathways [92]. Moreover, the decreased viability on the coated AR850 sample could be associated with the presence of a greater fraction of anatase in the oxide since it was found that dissolution of Ti ions from the rutile phase was one order of magnitude lower than that of anatase [35].

**Figure 12:**
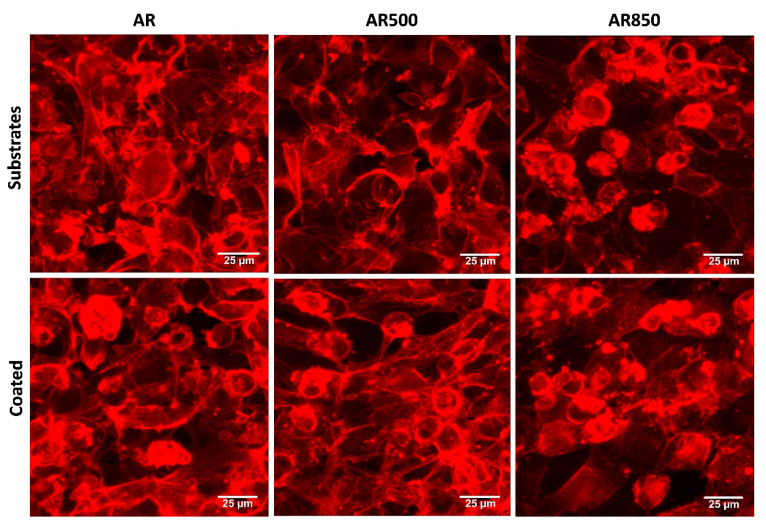
Representative fluorescent images showing cell morphology of MG63 cultured on bare and coated substrates for 24 h. The cytoskeleton was visualized by F-actin staining. Bars 25 µm.

The cell morphology of MG63 osteoblasts on each bare and coated surface was investigated by fluorescent microscopy following a cytoskeleton formation. Representative results are shown in Figure 12. The cells of the mechanically polished surface exhibited a flat phenotype typical for osteoblasts cultured on such surfaces [89], forming a monolayer. The cells attached to the coatings displayed polygonal elongated morphology extending from the main body filopodia and lamellipodia suggesting more differentiated physiology and direct focal contacts with the coating. A certain amount of filopodia was found for cells cultured on bare substrates but not as many as identified on the coated samples’ surface. Further, MG63 cell morphology was not abnormal when seeded on TiN/TiO_2_ coatings, suggesting that the nanostructured coatings were non-cytotoxic. After 24 h, cells on coated specimens exhibited long extensions of cytoplasmic membranes, making the cells fully spreading out the shape.

**Figure 13:**
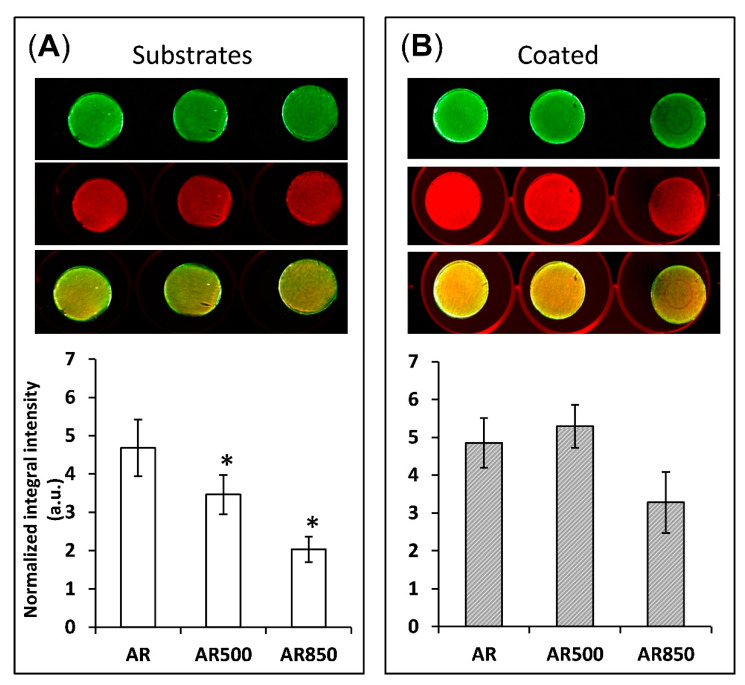
Bone mineralization activity of (**A**) uncoated and (**B**) coated samples after 31 days post-seeding of MG63 cells. * *p* < 0.01 compared to the AR sample. Mineralization was visualized by BoneTag CW800 (green) and cells by CellTag700 (red). * *p* < 0.05 compare to AR, n = 3.

The depth and width of grooves can determine the focal contacts of osteoblasts and the way they mineralize their matrix. For that reason, after 31 days of osteogenic induction, differentiation-induced calcium nodules on the surface of all samples were stained and measured by a quantitative colorimetric assay. The bone mineralization activity was found to be higher on the coated samples than on uncoated ones (Figure 13).

On the surface of EBM substrates (Figure 13A), osteoblasts tend to form congregates of calcium nodules in the cavities following the trajectory of the grooves. Similar behaviour of suppressed cell spreading on microroughened surfaces was also observed by Aita et al. [93]. However, even on the mechanically polished samples, calcium nodules were not homogeneously distributed on the surface (Figure 13A). In contrast, all coated samples displayed a more homogenous distribution of nodules on their surfaces. This fact indicates that the cells cultured on coated samples form focal attachments that allow them to spread across structures and build a homogenously mineralized matrix, especially on the coated AR500 samples. It follows that the coating boosted and enhanced cell differentiation. These favourable results can be explained by the relatively large material surface area available for cell adhesion, proliferation, differentiation and a change in spatial cell arrangement and protein adsorption. The present study indicates that the proposed hybrid treatment has a notable effect on biofilm formation like other surface modifying approaches referred to in [94]. It is generally considered that rapid bone mineral deposition on the surface of the implant results in earlier stabilization of the alloplastic material [95]. However, the changes that osteoblast cells undergo are complex and not easy to explain simply as a function of surface micro- and nanoarchitecture. For that reason, a limitation of our study is that we do not examine the profiles of gene expression and enzyme activity that control osteoclast differentiation as well as the relative rates of proliferation on each surface. Another limitation is that the influence of different rutile-to-anatase ratios in TiO_2_ should be clarified separately by samples with fixed weight percentages of both phases in the coatings. However, because of the naturally occurring processes that were earlier discussed, we consider coatings with changing rutile-to-anatase ratio while taking it into account in the present paper. Solving these two issues will be a task for our future studies.

## 4. Conclusions

By carefully adjusted experiments, EBM can be successfully used for surface hardening and regular surface roughening of Ti6Al4V alloy. After physical vapor deposition of 3.7 μm thick TiN/TiO_2_ film, the implant surfaces were found to offer high bond strength and bioactivity on the modified alloy. The coating formed on untreated alloy was richer in the rutile phase while an increase in anatase phase fraction was observed for those deposited on EBM Ti6Al4V alloy. The change in nitride texture and oxide phase composition of the coatings on EBM alloys decreased the nanohardness but further improved the COF and wear-resistance of the coatings. Additionally, all EBM and coated samples exhibited excellent corrosion resistance in PBS solution. Our results show that micro-dimensions and coating characteristics give important signals for osteoblast adhesion and bone mineral apposition. While cell attachment is dependent on micro-dimensions and spacing of the grooves, cell viability and calcium nodules deposition are highly influenced by sub-micron film features and its phase composition. The increased bone mineralization activity of osteoblast on the coated AR500 samples with sub-micron and microarchitecture suggests that synergetic mechanisms are involved. Considering the wear-resistance, anti-corrosion properties, and lack of cytotoxicity that hybrid surface treatment offers, it may be beneficial for improving the osseointegration of dental or orthopedic implants.

## Figures and Tables

**Table 1:** Main parameters and consistent deposition of the TiN/TiO_2_ multilayered film.

Process	Rotation (Hz)	Deposition Time (min)	Reactive Gas	Working Pressure (Pa)	Substrate Bias (-V)	Cathode Current (A)	Cathode Voltage (V)	Substrate Temp. (°C)
Cleaning	0.5	5	Ar	2.5 × 10^−1^	600	110	-	450
TiN	0.5	60	N_2_	7.5 × 10^−1^	250	110	-	340
TiO_2_	-	240	O_2_	6 × 10^0^	-	<1	1340	300

**Table 2:** Hardness and roughness values of TiAl6V4 substrate before and after treatment via different speed of sample motion (ν) and scanning frequency (f) of the electron beam.

ν (cm/s), f = 1 kHz	HV0.2	f (kHz), ν = 2 cm/s	HV0.2	Ra, (μm)
0.5 cm/s	364 ± 4.94	Unterated (polished)	323 ± 5.62	0.14 ± 0.004
1 cm/s	384.2 ± 4.98	500 Hz	405.4 ± 4.66	1.97 ± 0.045
1.5 cm/s	434.6 ± 4.62	650 Hz	392.8 ± 6.08	1.89 ± 0.046
2 cm/s	521 ± 5.67	750 Hz	400.2 ± 0.73	1.74 ± 0.051
2.5 cm/s	307.4 ± 1.83	850 Hz	387.5 ± 9.33	1.58 ± 0.050

**Table 3:** Statistical values of the surface roughness of TiN/TiO_2_ coatings deposited on the polished AR and AR500 samples: S_a_—average surface roughness, S_q_—root mean square roughness, S_sk_—skewness, R_pv_—average peak-to-valley roughness.

Sample	Scanned Area, μm	S_a_, (μm)	S_q_, (μm)	S_sk_	R_pv_, (μm)
Coated AR	50 × 50	0.624 ± 0.1	0.793 ± 0.2	0.479	5.736 ± 1.0
Coated AR500	50 × 50	1.726 ± 0.3	1.982 ± 0.3	0.018	8.789 ± 2.3
Coated AR850	50 × 50	1.080 ± 0.2	1.284 ± 0.1	−0.346	7.693 ± 1.9

**Table 4:** Hardness (H), elastic modulus (E), and their power-up ratios of TiN/TiO_2_ coatings deposited on AR and EBM Ti6Al4V alloy.

Sample	H, (GPa)	E, (GPa)	H^3^/E^2^
Coated AR	13.0 5 ± 2.07	279.55 ± 39.24	0.03
Coated AR850	9.02 ± 2.15	225.9 ± 85.2	0.01
Coated AR500	10.45 ± 2.17	262.02 ± 65.98	0.02

**Table 5:** Values of the average COF and wear track width after ball-on-wear tests for sliding time 300 s and adhesive critical for (Lc) determined by progressive scratch tests.

Sample	Average COF	Wear Track Width, (μm)	Adhesive Lc, (N)
AR	0.45 ± 0.04	233.7 ± 0.99	-
AR850	0.38 ± 0.06	223.3 ± 6.06	-
AR500	0.37 ± 0.05	221.3 ± 16.2	-
Coated AR	0.68 ± 0.16	552.4 ± 1.98	26.3 ± 1.3
Coated AR850	0.18 ± 0.02	216.5 ± 2.64	27.4 ± 1.2
Coated AR500	0.18 ± 0.03	215.5 ± 4.29	32.5 ± 3.5

**Table 6:** Representative electrochemical parameters derived from the respective polarization curves of the samples.

Sample	β_a_ (mV/dec)	-β_c_ (mV/dec)	E_corr_ (mV vs. Ag/AgCl)	j_corr_ (nA.cm^−2^)	j_pass_ * (μA.cm^−2^)	R_p_ (MΩ)
AR	171	194	−492	24	3.77	7.7
AR850	7	85	−101	8	2.69	20.1
AR500	544	74	−182	4.6	1.48	26
Coated AR	339	108	−74	62	0.27	2.1
Coated AR850	269	93	−70	68	0.26	2.6
Coated AR500	283	104	−68	54	0.25	2.8

* The passive current density was taken at 1 V.

## Data Availability

The data presented in this study are available on request from the corresponding author.

## Supplementary Materials

The following are available online at https://www.mdpi.com/article/10.3390/ijms22126369/s1.
