# Supplementary material for: Electrochemical, Tribological and Biocompatible Performance of Electron Beam Modified and Coated Ti6Al4V Alloy
Source: Int J Mol Sci. 2021 Jun 14;22(12):6369. doi: 10.3390/ijms22126369 (PMC8232333; doi:10.3390/ijms22126369)
Supplement: Supplementary file 1 [file ijms-22-06369-s001.zip › ijms-1232624-supplementary.pdf]

Supplementary

S 2.1. Equations used in XRD calculations

The texture coefficient (TC) for each (hkl) reflection was calculated after subtraction of background radiation by equation (1) [1]:

$$TC(hkl)=\frac{I_{(hkl)}/Io_{(hkl)}}{\frac{1}{n}\sum I_{(hkl)}/Io_{(hkl)}} \quad (1)$$

where $I_{(hkl)}$ and $I_{O\,(hkl)}$ are the measured diffraction intensity of the experimental spectra and powder pattern in JCPDS files, respectively, and n is the number of reflection peaks. For random distribution, TC is equal to 1.

The weight fractions of rutile (W_r) and anatase (W_a) phases in the coatings were determined by formula 2 and 3 [2].

$$W_r = \frac{I_r}{KI_a+I_r} \quad (2)$$

$$W_a = \frac{KI_a}{KI_a+I_r} \quad (3)$$

where Ir and Ia are the integrated intensities of (110) and (101) planes of the rutile and anatase phase. K is a coefficient equal to 0.886.

The grain size (D) was determined by the Debye-Scherrer equation (4):

$$D = \frac{0.9\lambda}{\beta Cos\theta} \quad (4)$$

where λ is the X-ray wavelength of Cu K_α radiation (1.5406 Å) and β is the full width at half maximum (in radian) and θ is Bragg's angle. Dislocation density (δ) which represents the number of defects in the films was calculated by using equation 5 [3]:

$$\delta = \frac{15\beta Cos\theta}{4aD} \quad (5)$$

where a is the cell parameter, and D is the grain size.

S 3.1

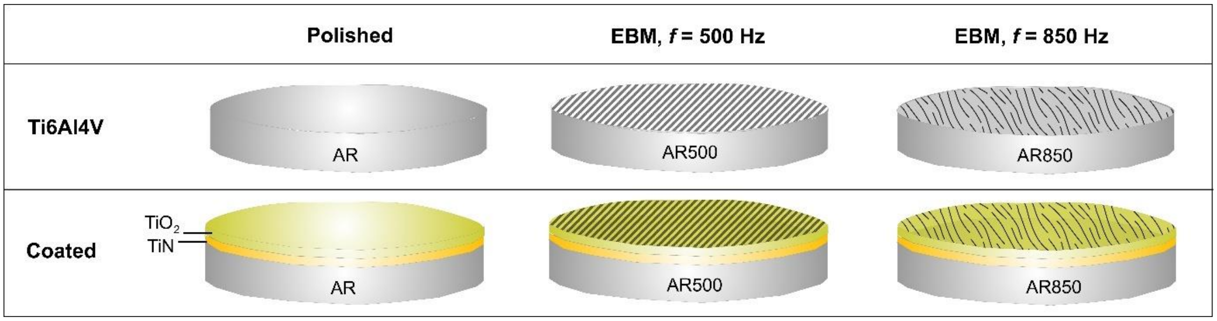

Figure S1. A schematic view of the specimens together with the samples' codification.

Figure S1 presents an overall view of the samples that have been examined in this study. After polishing, Ti6Al4V samples have been subjected to EBM with different electron beam frequencies (f = 500 and 850 Hz) and their surface roughness became different. The polished and EBM

specimens were coated with PVD deposited coatings consisting of thicker underlying TiN and thinner overlaying TiO_2.

S 3.4.

Table S1. Texture coefficients of different (hkl) reflections of the substrates and coatings.

(hkl)	TC(hkl)		
	AR	AR500	AR850
	α-Ti		
(100)	2.27	1.43	0.61
(002)	0.32	1.85	2.44
(101)	0.77	2.10	1.66
(102)	0.22	1.32	0.33
(110)	0.52	0.94	0.24
(103)	0.13	0.80	0.11
(201)	2.77	3.42	1.61
	Titanium nitride		
(111)	0.02	0.01	0.01
(200)	0.82	1.93	2.07
(220)	2.16	0.79	0.92
	Titanium Oxide – phase %		
R (110)	72.9	65.8	57.3
A (101)	27.1	34.1	42.7

Table S2. Average grain sizes and dislocation density of α-Ti, TiN, and TiO_2 calculated by using Scherrer's equation.

Phase	Reflection	Grain size, (nm)			Dislocation density, ($\times 10^{-14}$ lines m^{-2})		
		AR	AR500	AR850	AR	AR500	AR850
α-Ti	D_{100}	221.29	161.70	155.65	33	62	67
	D_{101}	197.16	159.22	153.34			
TiN	D_{200}	97.90	105.42	105.41	111	96	96
	D_{220}	71.51	72.89	75.77	176	159	157
TiO_2	Anatase-D_{101}	165.62	187.23	187.25	49	37	37
	Rutile-D_{110}	178.63	186.42	178.63	34	31	34

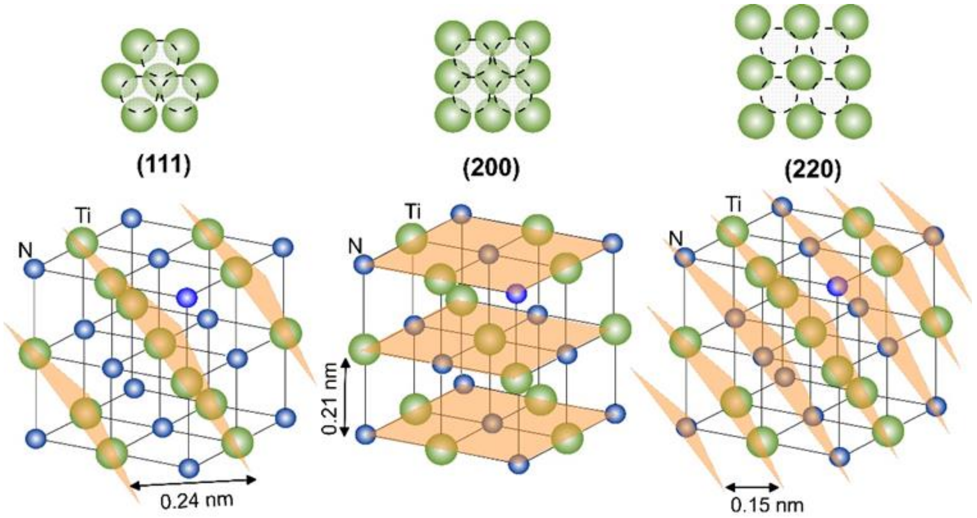

Figure S2. Crystallographic planes with their atomic arrangement and inter-planer distances in TiN phase.

S 3.5

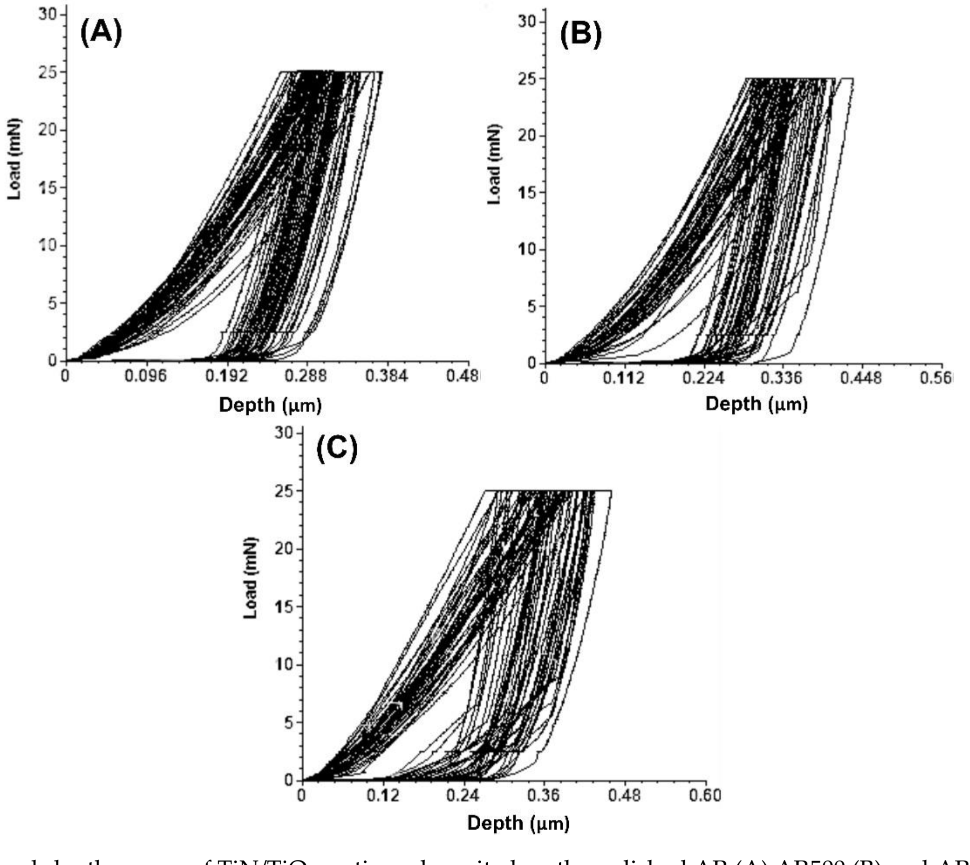

Figure S3. Load-depth curves of TiN/TiO_2 coatings deposited on the polished AR (A) AR500 (B) and AR850 (C) samples.

References

1. M. Hashemzadeh, K. Raeissi, F. Ashrafizadeh, S. Khorsand, Effect of ammonium chloride on microstructure, super-hydrophobicity and corrosion resistance of nickel coatings. Surf. Coat. Technol. 283 (2015) 318–328. https://doi.org/10.1016/j.surfcoat.2015.11.008
2. H. Zhang, J.F. Banfield, Understanding polymorphic phase transformation behavior during growth of nanocrystalline aggregates: Insights from TiO_2, J. Phys. Chem. B. 104 (2000) 3481-3487. https://doi.org/10.1021/jp000499j
3. Y.P.V. Subbaiah, P. Prathap, K.T.R., Reddy, Structural, electrical and optical properties of ZnS films deposited by close-spaced evaporation, Appl. Surf. Sci. 253(5) (2006) 2409-2415. https://doi.org/10.1016/j.apsusc.2006.04.063
